# QUaRTM: A Quadcopter with Unactuated Rotor Tilting Mechanism capable of faster, more agile, and more efficient flight

**DOI:** 10.3389/frobt.2022.1033715

**Published:** 2022-10-21

**Authors:** Jerry Tang, Karan P. Jain, Mark W. Mueller

**Affiliations:** High Performance Robotics Lab, Department of Mechanical Engineering, University of California, Berkeley, Berkeley, CA, United States

**Keywords:** morphing quadcopter, agile, efficient, high-speed, mechanism design (MD), aerodynamics

## Abstract

We present QUaRTM – a novel quadcopter design capable of tilting the propellers into the forward flight direction, which reduces the drag area and therefore allows for faster, more agile, and more efficient flight. The vehicle can morph between two configurations in mid-air, including the untilted configuration and the tilted configuration. The vehicle in the untilted configuration has a higher pitch torque capacity and a smaller vertical dimension. The vehicle in the tilted configuration has a lower drag area, leading to a higher top speed, higher agility at high speed, and better flight efficiency. The morphing is accomplished without any additional actuators beyond the four motors of a quadcopter. The rigid connections between the quadcopter frame and the quadcopter arms are replaced with sprung hinges. This allows the propellers to be tilted when high thrusts are produced, and recover to the untilted configuration when the thrusts are brought low. The effectiveness of such a vehicle is demonstrated by running experiments on a prototype vehicle with a shape similar to a regular quadcopter. Through the use of tilting, the vehicle is shown to have a 12.5% higher maximum speed, better high-speed agility as the maximum crash-free cruise speed increased by 7.5%, and a better flight efficiency as the power consumption has dropped by more than 20% in the speed range of 15–20 m s^−1^.

## 1 Introduction

Over the past decade, UAVs have become increasingly popular. One of the most common UAV designs is the quadcopter which is a multirotor device driven by four independently controlled propellers. The simplicity and agility of quadcopters as explained in (Mueller et al., 2022) have made them one of the preferred choices for a variety of applications, such as surveillance (Jaimes et al., 2008), mapping (Siebert and Teizer, 2014), building inspection, photography, delivery (Thiels et al., 2015), and disaster management (Erdelj et al., 2017). Nevertheless, conventional quadcopters are usually not able to achieve a high top speed, nor are they able to fly efficiently at a high speed. This is related to the fact that a quadcopter has to tilt its body toward the forward flight direction to counter the drag. However, tilting the quadcopter body means that a larger area is now subject to air resistance, which in turn increases the burden on the propellers. In addition to limiting the top speed, this also reduces the flight efficiency and might cause the quadcopter to stall. For tasks such as search and rescue and rapid package delivery that are both time and cost sensitive, and still require the vehicle to have high agility and vertical take-off capacity, we see a demand for a quadcopter platform that is capable of efficient high-speed flight (Frachtenberg, 2019; Poikonen and Campbell, 2020).

Some work has been done on increasing the flight efficiency and endurance of quadcopters. A method for finding the optimal speed and sideslip angle of a multicopter was presented in Wu et al. (2022). An approach to extending endurance and range by docking secondary quadcopters carrying replacement batteries is shown in Jain et al. (2020a). An increase in flight time by using the battery in multiple stages has been demonstrated in Jain et al. (2020b). Solar-powered UAVs, which can potentially fly large distances, have been explored in Reinhardt et al. (1996).

Since the limit on the top speed of a conventional quadcopter often has to do with its inherent aerodynamic properties, a more fundamental design change is often required to improve the efficiency and flight speed. A common design that can achieve the said goals is the tilt-rotor design. A tilt-rotor allows the propellers to be tilted toward the flight direction without the need for tilting the main body, thereby reducing the area subject to wind. Several tilt-rotor quadcopter designs have been explored. A convertible prototype “Quad Tilt Rotor” capable of vertical takeoffs like a quadcopter, and high-speed flight like a fixed-wing UAV was presented in Lin et al. (2014). A control scheme to handle the flight mode conversion from a helicopter to a fixed-wing “Quad-TiltRotors” was presented in Papachristos et al. (2013). A constrained robust model reference adaptive controller of an H-shaped tilt-rotor was presented in Anderson et al. (2021). In addition to fusing a fixed-wing and a conventional quadcopter to enable the vehicle to travel at a high speed, several other tilt-rotor designs have been explored. A tilt-rotor quadcopter capable of achieving any arbitrary desired state or configuration by tilting each rotor independently was presented in Nemati et al. (2016). The design and optimal control of an omnidirectional micro aerial vehicle capable of exerting a wrench in any orientation while maintaining efficient flight configurations were presented in Allenspach et al. (2020).

**FIGURE 1:**
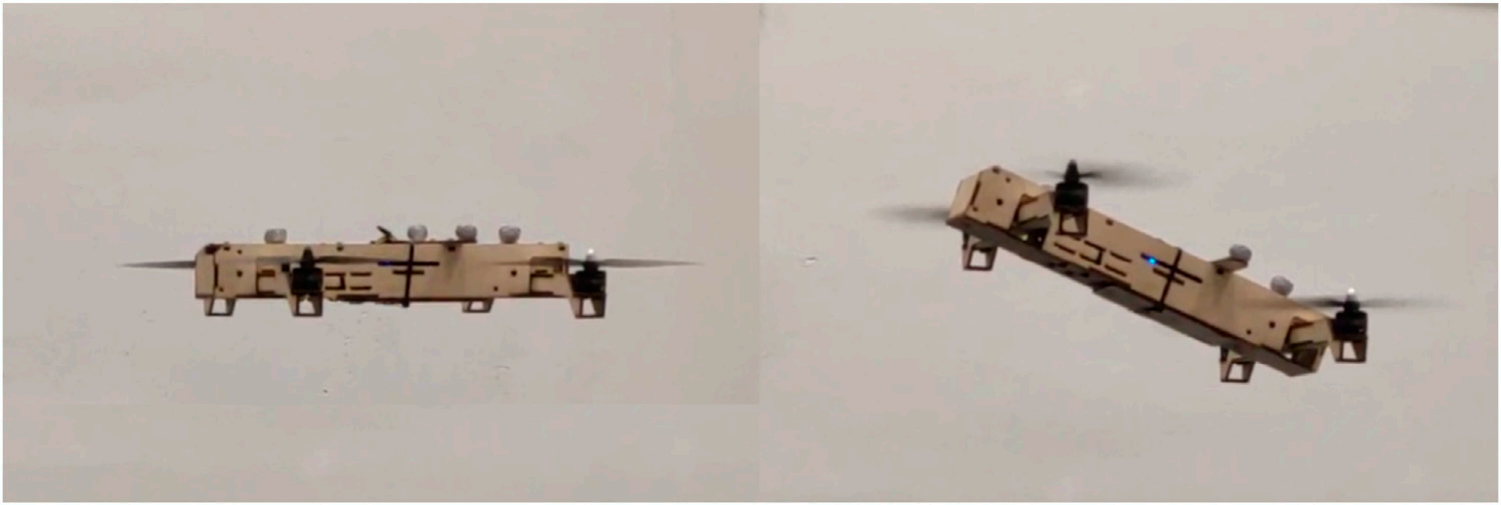
The experimental vehicle hovering in the untilted (left) and tilted (right) configurations.

**FIGURE 2:**
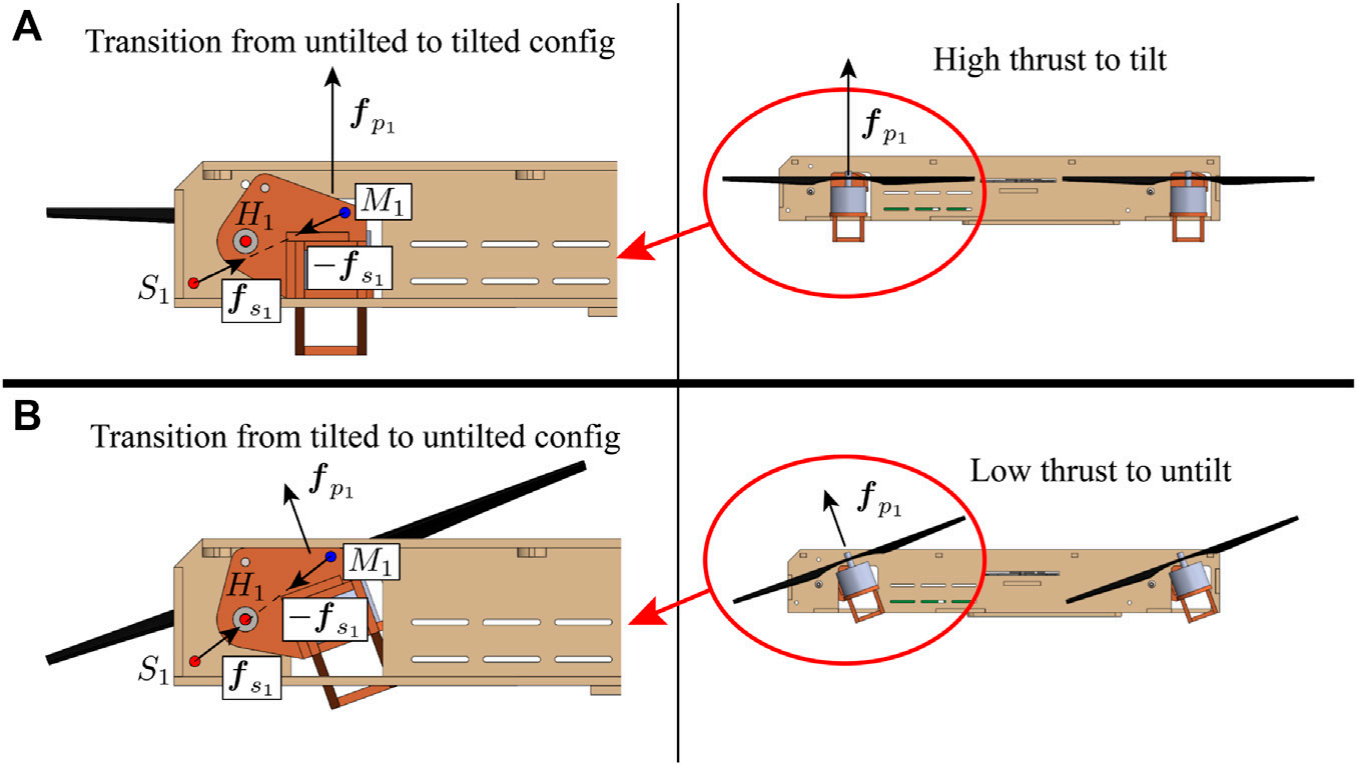
The vehicle viewing from the left side and the internal tilting mechanism for the front-right arm. **(A)** shows the vehicle in the untilted configuration, and **(B)** shows the vehicle in the tilted configuration. The fuselage is shown in beige. The arm shown in red orange is allowed to rotate around hinge *H*_1_. A spring is attached between point *S*_1_ on the quadcopter central body and *M*_1_ on the arm. The spring produces a torque on the arm around hinge *H*_1_ that tries to keep the arm in the untilted configuration. The tensions in the spring are very close for the two configurations because the amount of extension is nearly identical. However, in the untilted configuration **(A)**, the distance from hinge *H*_1_ to line *S*_1_ *M*_1_ is large, leading to a large moment arm and a large torque produced by the spring. Thus the arm will only tilt when a very high thrust is produced, allowing the vehicle in the untilted configuration to operate at a high maximum thrust. Once the spring torque is overcome by producing a high thrust, the arm will tilt and the vehicle will transition to the tilted configuration **(B)**. The torque exerted by the spring will reduce because the moment arm has reduced. This ensures that the arm will not untilt so easily, allowing the vehicle in the tilted configuration to operate at a low minimum thrust.

We propose a novel tilt-rotor vehicle design – a quadcopter with an unactuated rotor tilting mechanism (QUaRTM), capable of tilting the propellers into the forward flight direction without the use of any actuators beyond the four quadcopter motors. QUaRTM has two configurations: the untilted configuration with all propeller planes parallel to the central body, and the tilted configuration with all rotors tilted into the forward flight direction by an angle of 20°. A photo of QUaRTM hovering in both configurations is shown in Figure 1. In contrast to a conventional quadcopter, the rigid connections between the quadcopter arms and the central body are replaced with hinges. This allows the propellers to tilt into the forward flight direction without having to tilt the central body. Springs are added at the hinges to pull the arms into the untilted configuration. When the net propeller thrust is high enough to overcome the torque from the springs, the vehicle will transition into the tilted configuration. Then from the tilted configuration, when the net propeller thrust drops below a threshold, the arms will untilt and restore the vehicle to the untilted configuration. The spring torque acting on the arm is high in the untilted configuration and low in the tilted configuration. This creates a mechanical hysteresis that *1*) prevents oscillations in the tilting behavior, *2*) avoids unintended tilting or untilting, and *3*) allows the propellers to produce a wider range of thrusts in both configurations. Figure 2 shows the internal architecture of the tilting mechanism.

QUaRTM thus combines both the advantages of flying in the untilted configuration and flying in the tilted configuration with some trade-offs. When flying in the untilted configuration, the offset between the front and rear rotors’ thrust axes is the largest, resulting in the highest pitch torque capacity at lower speed. In addition, since the propellers’ plane is parallel to the quadcopter frame’s top plane, the vertical dimension of the vehicle is small, which makes it theoretically possible for the vehicle to fly through narrower gaps. When flying in the tilted configuration, since the central body is not tilted toward the forward flight direction, the drag reflected on the vehicle is low. This allows the vehicle to achieve a higher top speed and a higher energy efficiency. In addition, the reduction in drag allows a greater portion of the vehicle’s thrust capacity to be used for maneuvering instead of merely countering drag. This improves the high-speed agility of the vehicle. On the other hand, this vehicle has a slightly reduced range of thrust and torques. This is because to prevent unintended tilting and untilting, additional thrust constraints on the propellers need to be imposed. In addition, there is a slight increase in the mass and mechanical complexity of the vehicle due to the addition of the tilting mechanism.

Therefore, we argue that the proposed design is advantageous to existing solutions where the quadcopter is primarily expected to take off and land vertically, and fly at a high speed with high agility. Such applications are common when the targets are time-sensitive, e.g. long-distance package delivery, drone racing, search and rescue. This paper will discuss the dynamics of QUaRTM, the principles that govern the design of the vehicle, the experimental vehicle and its controller, and the experiments conducted to validate the design and its capabilities, including *1*) the mid-air tilting and untilting transitions, *2*) the improvements on the maximum vehicle speed and high-speed agility, and *3*) the improvements on flight efficiency when the vehicle travels at a high speed.

## 2 System overview

In this section, we will provide an overview of the system. We will define the model of the vehicle and derive its dynamics. This will help us to *1*) find the constraints on the propeller thrusts to prevent unintended tilting and untilting, and *2*) design for the vehicle frame and the tilt angle.

### 2.1 Notation

We follow the notations in (Bucki and Mueller, 2019) for defining the model of the vehicle. Non-bold symbols like *m* represent scalars, lowercase bold symbols like ***g*** represent vectors, and uppercase bold symbols like ***J*** represent matrices. Subscripts such as *m*_*C*_ represent the body to which the symbol refers, and superscripts such as ***g*** ^*E*^ represent the frame in which the vector is expressed. A second subscript or superscript such as ***ω***_*CE*_ or ***R*** ^*CE*^ represents what the quantity is defined with respect to. However, the special superscript ^*T*^ represents the transpose of a matrix. To express a cross product, we use the skew-symmetric matrix form such that ***a*** × ***b*** = ***S*** (***a***) ***b***. The symbol ***d*** represents a displacement, ***ω*** represents an angular velocity, and ***R*** represents a rotational matrix.

### 2.2 Model

**FIGURE 3:**
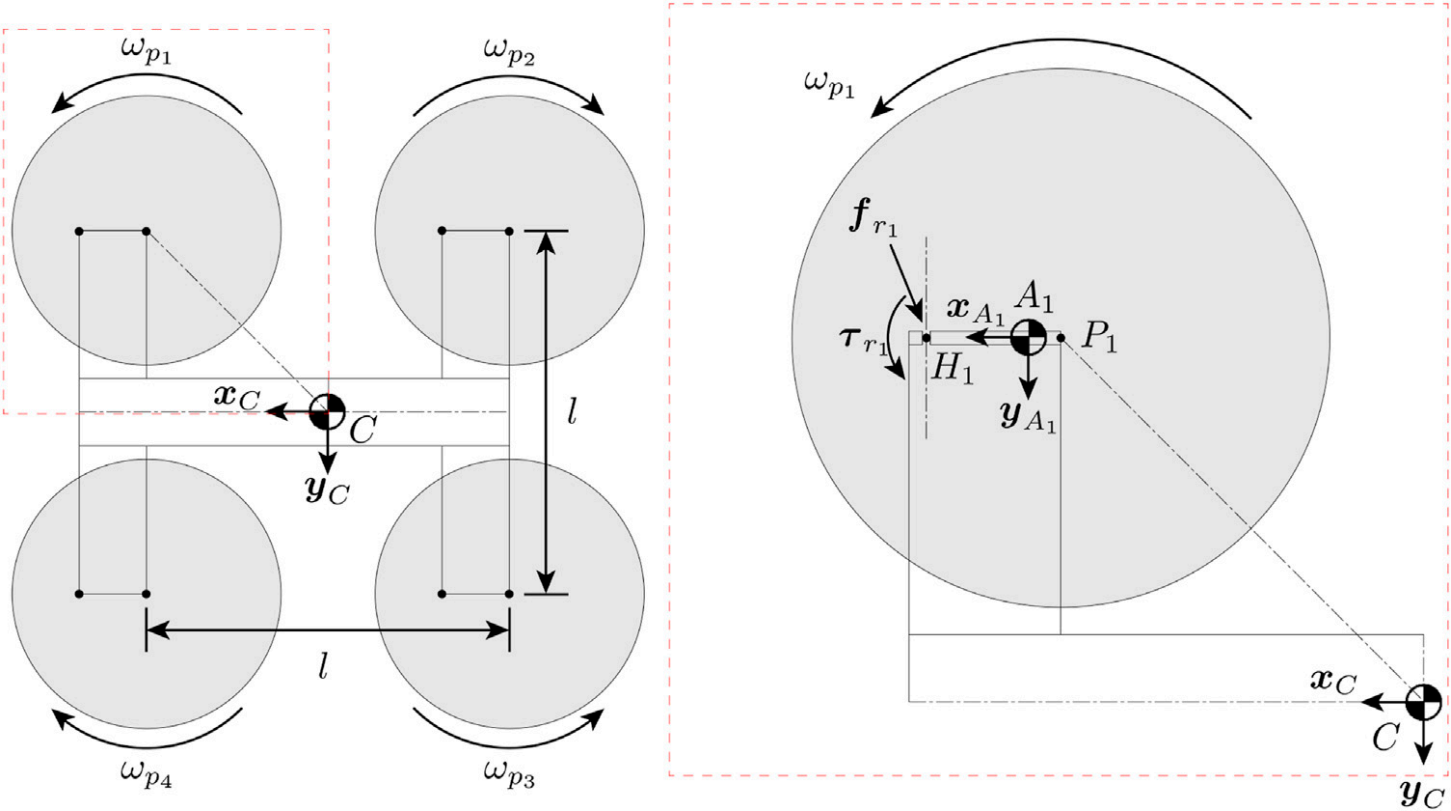
Top-down view of the vehicle model in the untilted configuration. The propellers are numbered 1, 2, 3, 4 in a clockwise manner. The right side shows the detailed view of rotor 1, where *P*_1_ is the location of the rotor, *A*_1_ is the COM of the arm that the rotor is attached to, and *H*_1_ is the hinge that the arm can tilt about. Reaction force $f_{r_{1}}$ and torque $\tau_{r_{1}}$ act in opposite directions between each arm and the central body at the hinge *H*_1_.

First of all, we define a model of the vehicle which we will use for analysis. Figure 3 shows the quadcopter model as seen from the top. We model the system as five coupled rigid bodies, including the central body of the quadcopter and the four quadcopter arms with the rotors mounted. We denote the Earth frame as *E*, the central body frame as *C*, and the frame for each arm as *A*_*i*_ for *i* ∈ {1, 2, 3, 4}. The origin of any frame is defined to be at the center of mass of the corresponding body. For the central body frame, the *x*-axis ***x***_*C*_ points to the front of the vehicle, and the *z*-axis ***z***_*C*_ points upward from the body’s top surface. The rotation matrix of central body frame *C* with respect to the Earth frame *E* is defined as ***R*** ^*CE*^. For a vector expressed in the Earth frame ***v*** ^*E*^, ***v*** ^*C*^ = ***R*** ^*CE*^ ***v*** ^*E*^ represents its expression in the central body frame.

Each arm is allowed to tilt with respect to the central body frame *C* around the ***y***_*C*_ direction, and the fully tilted tilt angle is defined as *β*. Throughout this paper, we will assume that all arms tilt at the same angle. We also define the combined arm frame *A* which has axes aligned with any arm *i*, and its origin located at the center of mass of the whole vehicle. When an arm is not tilted, all three axes point in the same directions as those of the central body frame, that $x_{C}=x_{A_{i}},y_{C}=y_{A_{i}},z_{C}=z_{A_{i}}$ . Since tilting only happens in the $y_{C}=y_{A_{i}}$ direction, only $z_{A_{i}}$ and $x_{A_{i}}$ will change when the arm tilts. The rotation matrix of an arm with respect to the central body is thus a single degree of freedom rotation matrix defined as $R^{A_{i}C}$ .

**FIGURE 4:**
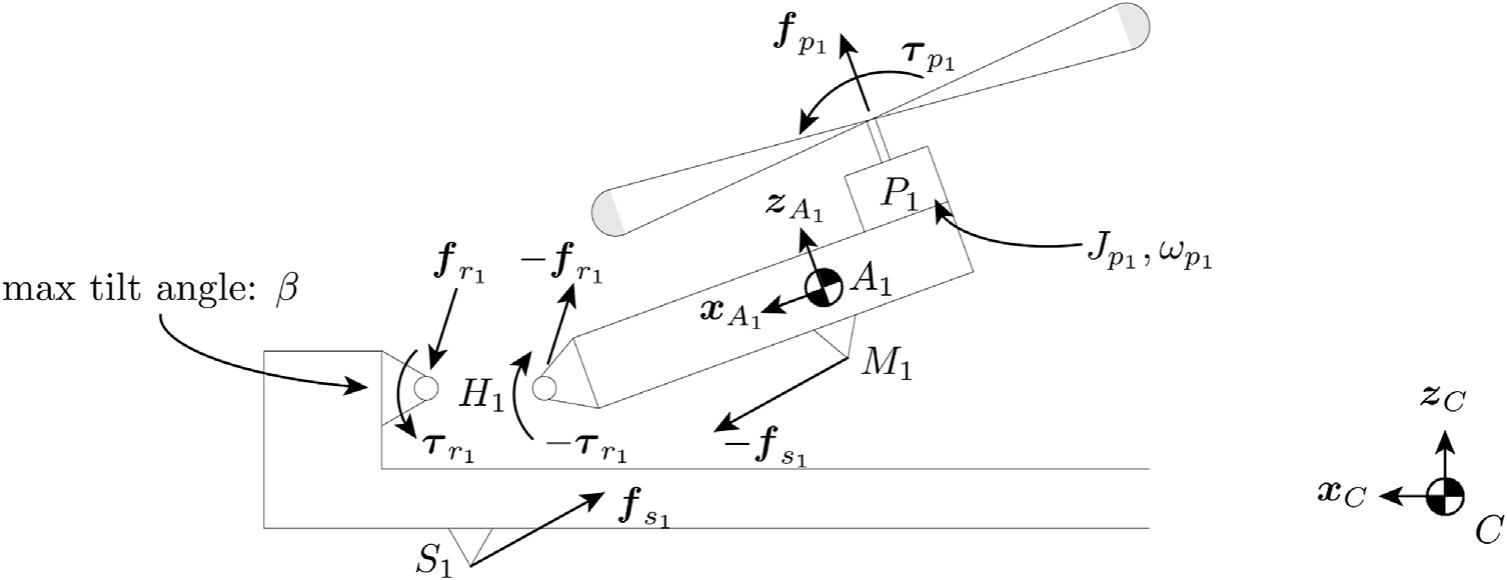
Free-body diagram showing the forces acting on arm 1 viewing from the left side. The length of the arm is exaggerated. A spring is attached between point *S*_1_ on the quadcopter central body and *M*_1_ on the arm. The spring exerts a torque that tries to keep the arm in the untilted configuration. The propeller produces a thrust force $f_{p_{i}}$ and a torque $\tau_{p_{i}}$ in the $z_{A_{1}}$ direction. The momentum of inertia of the rotor around its axis is $J_{p_{1}}$ , and the rotor rotates at a speed of $\omega_{p_{1}}$ .

Figure 4 shows forces and torques acting on arm 1. Note that while the figure shows only arm 1, the model can be generalized to all arms. To control the thrust at which the arm will tilt or untilt, a spring producing a force $f_{s_{i}}$ is connected between point *S*_*i*_ on the central body and point *M*_*i*_ on arm *i*. Note that spring is not the only option here but rather a design choice. Other widgets like magnets can also be used to produce such force. In addition to the spring force and the total acceleration force, arm *i* also sees the propeller force and torque $\left(f_{p_{i}}=f_{p_{i}}z_{p_{i}},\tau_{p_{i}}=\tau_{p_{i}}z_{p_{i}}\right)$ , and the hinge’s reaction force and torque $\left(-f_{r_{i}},-\tau_{r_{i}}\right)$ . The mass and moment of inertia of the central body at its center of mass are denoted as *m*_*C*_ and ***J***_*C*_ respectively. Similarly, the mass and moment of inertia of any arm *i* at its center of mass are denoted as *m*_*A*_ and ***J***_*A*_.

### 2.3 Aerodynamics

Now, we model the aerodynamics of the vehicle. We will use these results to design for the tilt angle in Section 3.3. We express the aerodynamics of the quadcopter in the Earth frame *E*. Assuming that the quadcopter is cruising in the ***x***_*E*_ direction at a fixed height, the drag and lift forces are:

$$f_{D}=-\frac{1}{2}C_{D}\left(\alpha \right)\rho Av^{2}x_{E}$$ (1)

$$f_{L}=\frac{1}{2}C_{L}\left(\alpha \right)\rho Av^{2}z_{E}$$ (2)

**FIGURE 5:**
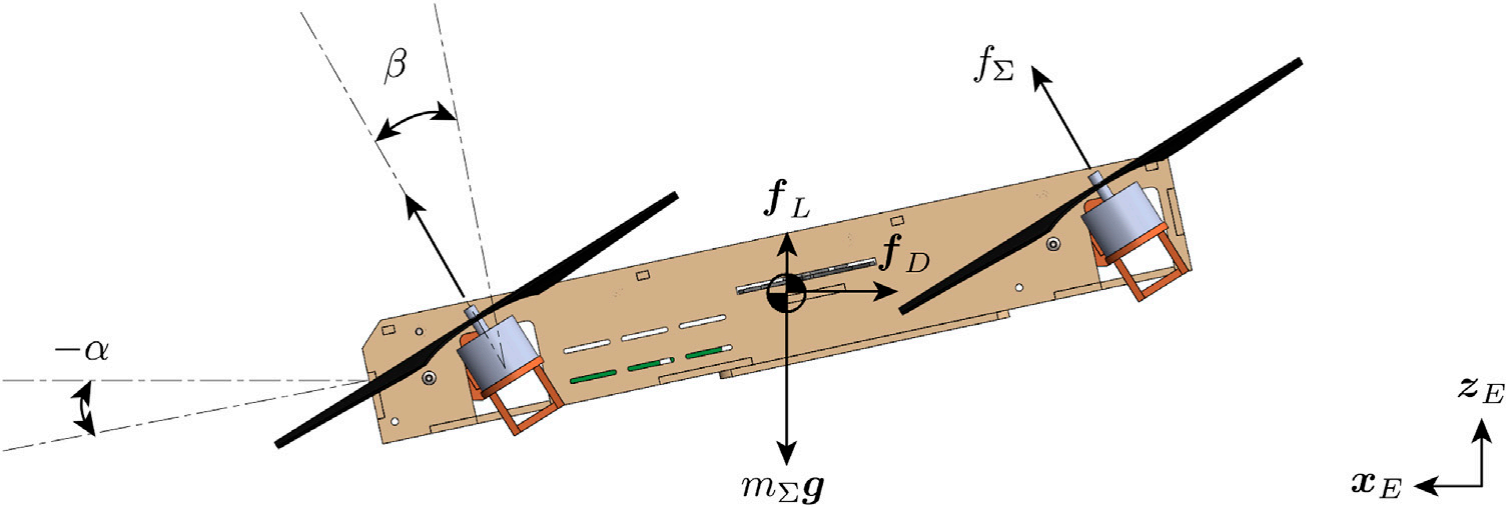
Free-body diagram of the vehicle when cruising. The tilt angle *β* is the angle between the arm and the central body, and the angle of attack *α* is the angle between the central body and the horizon.

Where *α* is the angle of attack, *C*_*D*_(*α*) and *C*_*L*_(*α*) are the angle-of-attack-dependent drag and lift coefficients, *ρ* is the density of air, *v* is the speed of the quadcopter, and *A* is the reference area which is the projection area of the vehicle onto its top surface. Figure 5 shows the breakdown of forces on the quadcopter when it is cruising. The force balance of the quadcopter can be expressed as:

$$f_{\Sigma }\sin\left(-\alpha +\beta \right)=\frac{1}{2}C_{D}\left(\alpha \right)\rho Av^{2}$$ (3)

$$f_{\Sigma }\cos\left(-\alpha +\beta \right)=m_{\Sigma }g-\frac{1}{2}C_{L}\left(\alpha \right)\rho Av^{2}$$ (4)

Where $f_{\Sigma }≔\sum_{i=1}^{4}f_{p_{i}}$ is the total thrust from all four propellers, *m*_Σ_ is the total mass of the vehicle, and *g* is the gravitational acceleration. We will use the results here to design for the tilt angle in Section 3.3.

### 2.4 Rigid body dynamics

We derive the rigid body dynamics of the vehicle, especially those governing the tilting of the arms. We will use these results in Section 3.1 to design for the vehicle configuration, and in Section 4.1 to compute the bounds on the control inputs to ensure that mid-air morphing happens in a controlled manner. Since tilting and untilting usually happen during the early and late stages of flight where the speed is low, we will not consider aerodynamic forces here. The translational and rotational dynamics of the vehicle can be computed using Netwon’s and Euler’s laws of motion Zipfel (2007). The translational dynamics of the central body expressed in the Earth frame *E*, and the rotational dynamics of the central body expressed in the central body frame *C* are:

$$m_{C}{\ddot{d}}_{CE}^{E}=m_{C}g^{E}+R^{EC}\overset{4}{\underset{i=1}{\sum }}\left(f_{r_{i}}^{C}+f_{s_{i}}^{C}\right)$$ (5)

$$J_{C}^{C}{\dot{\omega }}_{CE}^{C}+S\left(\omega_{CE}^{C}\right)J_{C}^{C}\omega_{CE}^{C}=\overset{4}{\underset{i=1}{\sum }}\left(\tau_{r_{i}}^{C}+S\left(d_{H_{i}C}^{C}\right)f_{r_{i}}^{C}+S\left(d_{CS_{i}}^{C}\right)f_{s_{i}}^{C}\right)$$ (6)

The translational and rotational dynamics of arm *i* expressed both in the corresponding arm frame are:

$$\begin{array}{ll} m_{A_{i}}R^{A_{i}E}{\ddot{d}}_{CE}^{E} & =-m_{A_{i}}R^{A_{i}C}\left(S\left(d_{CH_{i}}^{C}\right){\dot{\omega }}_{CE}^{C}+S\left(\omega_{CE}^{C}\right)d_{CH_{i}}^{C}\omega_{CE}^{C}\right)\\ & \;-m_{A_{i}}\left(S\left(d_{H_{i}A_{i}}^{A_{i}}\right){\dot{\omega }}_{A_{i}E}^{A_{i}}+S\left(\omega_{A_{i}E}^{A_{i}}\right)S\left(d_{H_{i}A_{i}}^{A_{i}}\right)\omega_{A_{i}E}^{A_{i}}\right)\\ & \;+z_{A_{i}}^{A_{i}}f_{p_{i}}-f_{r_{i}}^{A_{i}}-f_{s_{i}}^{A_{i}}+m_{A_{i}}R^{A_{i}E}g^{E} \end{array}$$ (7)

$$\begin{array}{ll} J_{A_{i}}^{A_{i}}{\dot{\omega }}_{A_{i}E}^{A}+S\left(\omega_{A_{i}E}^{A_{i}}\right)J_{A_{i}}^{A_{i}}\omega_{A_{i}E}^{A_{i}} & =S\left(d_{P_{i}A_{i}}^{A_{i}}\right)z_{A_{i}}^{A_{i}}f_{p_{i}}+z_{A_{i}}^{A_{i}}\tau_{p_{i}}-\tau_{r_{i}}^{A_{i}}-S\left(d_{H_{i}A_{i}}^{A_{i}}\right)f_{r_{i}}^{A_{i}}\\ & \;-S\left(d_{M_{i}A_{i}}^{A_{i}}\right)f_{s_{i}}^{A_{i}}-J_{p_{i}}S\left(\omega_{A_{i}E}^{A_{i}}\right)\omega_{p_{i}}z_{A_{i}}^{A_{i}} \end{array}$$ (8)

Where $J_{p_{i}}$ is the moment of inertia of the rotor, $\omega_{p_{i}}$ is the rotational speed of the propeller, and the last term $J_{p_{i}}S\left(\omega_{A_{i}E}^{A_{i}}\right)\omega_{p_{i}}z_{A_{i}}^{A_{i}}$ indicates the gyroscopic torque produced by rotating the spinning rotor.

Now we consider the dynamics of the whole quadcopter. Its translational dynamics in the Earth frame *E* and rotational dynamics in the central body frame *C* are:

$$m_{\Sigma }{\ddot{d}}_{CE}^{E}=m_{\Sigma }g^{E}+R^{EC}z_{A_{i}}^{C}\overset{4}{\underset{i=1}{\sum }}f_{p_{i}}=m_{\Sigma }g^{E}+R^{EC}z_{A_{i}}^{C}f_{\Sigma }$$ (9)

$$J_{\Sigma }^{C}{\dot{\omega }}_{CE}^{C}+S\left(\omega_{CE}^{C}\right)J_{\Sigma }^{C}\omega_{CE}^{C}=\overset{4}{\underset{i=1}{\sum }}S\left(d_{P_{i}C}^{C}\right)z_{A_{i}}^{C}f_{p_{i}}+z_{A_{i}}^{C}\tau_{p_{i}}=\tau_{\Sigma }^{C}$$ (10)

Where ***J***_Σ_ is the moment of inertia of the whole quadcopter, and ***τ***_Σ_ is the net torque produced by the four propellers on the quadcopter. We can use these equations to compute the linear and angular accelerations of the quadcopter:

$${\ddot{d}}_{CE}^{E}=g^{E}+\frac{1}{m_{\Sigma }}R^{EC}z_{A_{i}}^{C}f_{\Sigma }$$ (11)

$${\dot{\omega }}_{CE}^{C}=J_{\Sigma }^{C-1}\tau_{\Sigma }^{C}-S\left(\omega_{CE}^{C}\right)J_{\Sigma }\omega_{CE}^{C}$$ (12)

Finally, plugging these equations back into the dynamics of the arm, we can find the reaction force $f_{r_{i}}^{A_{i}}$ and torque $\tau_{r_{i}}^{A_{i}}$ acting at the hinge:

$$f_{r_{i}}^{A_{i}}=m_{A}\left(R^{A_{i}E}\left(g^{E}-{\ddot{d}}_{CE}^{E}\right)-S\left(d_{CA_{i}}^{A_{i}}\right)R^{A_{i}C}{\dot{\omega }}_{CE}^{C}\right.$$

$$\left.\;+R^{A_{i}C}\left(S\left(\omega_{CE}^{C}\right)S\left(d_{CH_{i}}^{C}\right)\omega_{CE}^{C}+S\left(\omega_{A_{i}E}^{C}\right)S\left(d_{H_{i}A_{i}}^{C}\right)\omega_{A_{i}E}^{C}\right)\right)+z_{A_{i}}^{A_{i}}f_{p_{i}}-f_{s_{i}}^{A_{i}}$$ (13)

$$\begin{array}{ll} \tau_{r_{i}}^{A_{i}} & =R^{A_{i}C}\tau_{p_{i}}^{C}-S\left(d_{M_{i}A_{i}}^{A_{i}}\right)f_{s_{i}}^{A_{i}}-S\left(d_{H_{i}A_{i}}^{A_{i}}\right)f_{r_{i}}^{A_{i}}-J_{A_{i}}^{A_{i}}R^{A_{i}C}{\dot{\omega }}_{CE}^{C}\\ & \;-R^{A_{i}C}S\left(\omega_{A_{i}E}^{C}\right){\left(R^{A_{i}C}\right)}^{T}J_{A_{i}}^{A_{i}}R^{A_{i}C}\omega_{A_{i}E}^{C}+J_{p_{i}}S\left(\omega_{A_{i}E}^{A_{i}}\right)\omega_{p_{i}}z_{A_{i}}^{A_{i}} \end{array}$$ (14)

We note that for the arm to remain untilted, the hinge should only apply a negative reaction torque on the arm. Similarly, for the arm to remain tilted, the hinge should only apply a positive reaction torque on the arm. In math form, $y_{A_{i}}^{A_{i}}\cdot \tau_{r_{i}}^{A_{i}}\leq 0$ if the arm is to remain untilted, and $y_{A_{i}}^{A_{i}}\cdot \tau_{r_{i}}^{A_{i}}\geq 0$ if the arm is to remain tilted. We note that this constraint only holds when the arms tilt independently. However, the tilting of the arms could be coupled mechanically to relax the bounds. There are three arm coupling configurations. The first is the non-coupled configuration, where each arm tilts separately from one another. The second is the side-coupled configuration, where the two arms at the front are coupled and the two arms at the back are coupled, or the two arms on the left are coupled and the two arms on the right are coupled. The third is the all-coupled configuration, where all four arms are coupled to rotate together. The thrust bounds thus become:

$$\text{Non}-\text{coupled}:y_{A_{i}}^{A_{i}}\cdot \tau_{r_{i}}^{A_{i}}\leq 0,\text{for }i\in \left\{1,2,3,4\right\}$$ (15)

$$\text{Side}-\text{coupled}:\left\{\begin{array}{c} y_{A_{1}}^{A_{1}}\cdot \tau_{r_{1}}^{A_{1}}+y_{A_{4}}^{A_{4}}\cdot \tau_{r_{4}}^{A_{4}}\;\leq 0\;\\ y_{A_{2}}^{A_{2}}\cdot \tau_{r_{2}}^{A_{2}}+y_{A_{3}}^{A_{3}}\cdot \tau_{r_{3}}^{A_{3}}\;\leq 0\; \end{array}\right.,\text{or}\left\{\begin{array}{c} y_{A_{1}}^{A_{1}}\cdot \tau_{r_{1}}^{A_{1}}+y_{A_{2}}^{A_{2}}\cdot \tau_{r_{2}}^{A_{2}}\;\leq 0\;\\ y_{A_{3}}^{A_{3}}\cdot \tau_{r_{3}}^{A_{3}}+y_{A_{4}}^{A_{4}}\cdot \tau_{r_{4}}^{A_{4}}\;\leq 0\; \end{array}\right.$$ (16)

$$\text{All}-\text{coupled}:\overset{4}{\underset{i=1}{\sum }}y_{A_{i}}^{A_{i}}\cdot \tau_{r_{i}}^{A_{i}}\leq 0$$ (17)

We will use the results here to evaluate the vehicle agility and choose the arm-coupling configuration in Section 3.1 and Section 3.2, and compute the bounds on the control inputs to ensure that mid-air morphing happens in a controlled manner in Section 4.1.

## 3 Design

In this section, we will discuss the design of the quadcopter. The key design parameters are the arm coupling configuration and the tilt angle. The arm coupling configuration affects vehicle agility. The tilt angle mainly affects the drag force, flight speed, and high-speed agility. We design our vehicle by first choosing an arm coupling configuration and designing an overall vehicle frame. Then, we will use the parameters of the vehicle frame to analyze the impact of the tilt angle on the vehicle performance and decide on the tilt angle.

### 3.1 Arm-coupling configuration and agility

For a conventional quadcopter, the only limits on the vehicle agility are the maximum and minimum thrusts and torques that a propeller can produce (*f*_min_, *f*_max_, *τ*_min_, *τ*_max_). For our vehicle, however, we need to impose additional bounds on the propeller thrusts to prevent the arms from tilting and untilting when not commanded to. These bounds are governed by the spring forces $f_{s_{i}}$ and some other dynamics effects as shown in Section 2.4.

To get a more intuitive understanding of how these bounds affect the agility of the vehicle and what we can do about it, let us consider a simplified case where the quadcopter is initially hovering in the untilted configuration, the angular acceleration is small, and the angular speed is small. $R^{A_{i}C}$ thus becomes identity, and all terms of vehicle angular acceleration and quadratic terms of vehicle angular velocity drop out. The reaction torque in the $y_{A_{i}}^{A_{i}}$ direction from Eq. 14 thus simplifies to:

$$\begin{array}{ll} y_{A_{i}}^{A_{i}}\cdot \tau_{r_{i}}^{A_{i}} & =-y_{A_{i}}^{A_{i}}\cdot \left(S\left(d_{M_{i}A_{i}}^{A_{i}}\right)+S\left(d_{A_{i}H_{i}}^{A_{i}}\right)\right)f_{s_{i}}^{A_{i}}+d_{H_{i}A_{i},x}^{A_{i}}\left(-m_{A}\frac{f_{\Sigma }}{m_{\Sigma }}+f_{p_{i}}\right)\\ & \;+y_{A_{i}}^{A_{i}}\cdot \left(J_{p_{i}}S\left(\omega_{A_{i}E}^{A_{i}}\right)\omega_{p_{i}}z_{A_{i}}^{A_{i}}\right) \end{array}$$ (18)

$$=d_{M_{i}H_{i},x}^{A_{i}}f_{s_{i},z}^{A_{i}}+d_{H_{i}A_{i},x}^{A_{i}}\left(-m_{A}\frac{f_{\Sigma }}{m_{\Sigma }}+f_{p_{i}}\right)-J_{p_{i}}\omega_{A_{i}E,x}^{A_{i}}\omega_{p_{i}}$$ (19)

Where $d_{M_{i}H_{i},x}^{A_{i}}f_{s_{i},z}^{A_{i}}$ represents the torque that the spring applies on the arm in the $y_{A_{i}}^{A_{i}}$ direction around the hinge, $d_{H_{i}A_{i},x}^{A_{i}}\left(-m_{A}\frac{f_{\Sigma }}{m_{\Sigma }}+f_{p_{i}}\right)$ represents the net torque from the thrust of the propeller and the inertial force from accelerating the arm around the hinge, and $J_{p_{i}}\omega_{A_{i}E,x}^{A_{i}}\omega_{p_{i}}$ represents the gyroscopic torque from the rotor. The propeller thrust just enough to tilt the arm is thus:

$$f_{p_{i},tilt}=m_{A}\frac{f_{\Sigma }}{m_{\Sigma }}-\frac{d_{M_{i}H_{i},x}^{A_{i}}}{d_{H_{i}A_{i},x}^{A_{i}}}f_{s_{i},z}^{A_{i}}+\frac{J_{p_{i}}\omega_{A_{i}E,x}^{A_{i}}\omega_{p_{i}}}{d_{H_{i}A_{i},x}^{A_{i}}}$$ (20)

The tilting behavior is thus determined by not only the spring-related parameters which the designer can decide, but also the roll motion of the vehicle due to the gyroscopic torque $J_{p_{i}}\omega_{A_{i}E,x}^{A_{i}}\omega_{p_{i}}$ . It turns out that the gyroscopic torque has a major negative impact on the agility of the vehicle. This is because the momentum of the rotor $J_{p_{i}}\omega_{p_{i}}$ is usually quite large due to the high rotational speed of the rotor, and its product with the roll speed of the quadcopter which gives the gyroscopic torque could easily exceed the torque from the spring that is holding the arm in place, which can result in unintended tilting and untilting. To reduce the impact of the gyroscopic torque on the tilting behavior, we will have to couple the rotations of two adjacent arms, and force them to tilt together. Since every two adjacent propellers spin in opposite directions, the net angular momentum will cancel out if the speeds are close, and will significantly reduce the gyroscopic torque reflected on the arms. The uncoupled configuration of Eq. 15 is thus not physically meaningful for most vehicles, and coupling the arms is always required. Near hover, because all rotors have similar speeds, coupling the arms essentially makes the gyroscopic torque drop out from the force balance, which will then further simplify the thrust to tilt the arm to:

$$f_{p_{i},tilt}=m_{A}\frac{4f_{p_{i},tilt}}{m_{\Sigma }}-\frac{d_{M_{i}H_{i},x}^{A_{i}}}{d_{H_{i}A_{i},x}^{A_{i}}}f_{s_{i},z}^{A_{i}}$$ (21)

The tilt thrust is now only related to the mechanical properties of the vehicle, and is thus a design parameter that we can choose. The same applies to the untilt thrust. Typically, we will want the tilt thrust to be large but smaller than the propeller’s maximum thrust, and the untilt thrust to be small but larger than the propeller’s minimum thrust. This will ensure a wide thrust range in either configuration and improves the agility of the vehicle. Near hover, the propeller thrust is thus bounded by $\left\{f_{\min},f_{p_{i},tilt}\right\}$ when the arms are untilted, and $\left\{f_{p_{i},untilt},f_{\max}\right\}$ when the arms are tilted. Once the desired tilt and untilt thrusts are set, the corresponding spring and anchoring points can be picked to generate the desired thrusts.

We do note that coupling the arms increases the complexity of the vehicle, as some external connecting rods may be required. However, we also note that the two arms at the front share the same axis for tilting, as well as the two arms at the back. Therefore, we can use a single arm to mount the two rotors at the front and at the back. Then we will only need to use one hinge and one spring to tilt each rotor pair. This will make the quadcopter H-shaped instead of X-shaped, and will eliminate the need for an external connecting rod.

**FIGURE 6:**
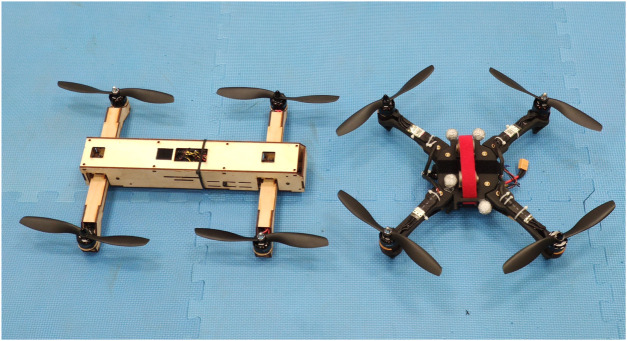
H-shaped (left) vs. X-shaped (right) quadcopter frame. Using the H-shaped frame means that a single hinge and a single spring can be used to tilt two propellers at the same time, which reduces the mechanical complexity of the vehicle.

Figure 6 shows an H-shaped vehicle and an X-shaped vehicle. While using the H-shaped frame increases the length of the quadcopter’s fuselage, having a longer fuselage makes the quadcopter more streamlined and thus more aerodynamically efficient. Considering the agility of the vehicle, it is recommended to use the side-coupled configuration for slow maneuvers, and use the all-coupled configuration for more agile maneuvers. An example of how a vehicle with the all-coupled configuration is more agile than a vehicle with the side-coupled configuration is provided later in Section 4.1. In addition, an H-shaped quadcopter frame is usually preferred in order to reduce mechanical complexity. Once the arm-coupling configuration is chosen, the overall vehicle frame can be designed. Next, the relevant parameters can be used to design for the tilt angle.

### 3.2 Experimental vehicle frame design

Following the ideas of Section 3.1, we decided to use the all-coupled configuration for our experimental vehicle, and developed an H-shaped vehicle frame. The properties of the experimental vehicle frame are given in Table 1. The overall size of the vehicle is designed to be similar to a commonly used quadcopter. The motors are the EMAX MT2208 brushless motors, and the propellers are 8’ 8045 ABS propellers. Both are commercially available. In order to avoid discharging the battery at a rate beyond the safety range, we set our cap on the individual propeller thrust at *f*_max_ = 4.5 N. The drag and lift coefficients are determined experimentally by flying the vehicle at various constant speeds and curve-fitting the measured lift and drag forces. The rotations of all four arms are synchronized by using a four-bar mechanism that links the front arm to the rear arm. The four-bar mechanism can also be removed to convert the vehicle to the side-coupled configuration. While the mass of the springs can be different depending on the tilt angle, we can reasonably expect the entire tilting mechanism to add a mass of 50 g, which is about 6 percent of the mass of the whole vehicle.

**TABLE 1:** Experimental vehicle frame properties.

Symbol	Parameter	Value
$m_{A_{i}}$	Individual arm mass	75 g
*m*_*C*_	Central body mass	550 g
*m*_Σ_	Total vehicle mass	850 g
*m*_*T*_	Tilting mechanism mass	50 g
*A*	Reference area	0.047m^2^
*C*_*D*_	Fitted drag coefficient equation	0.773*α* ^2^ + 0.543
*C*_*L*_	Fitted lift coefficient equation	1.264*α*
*l*	Distance between adjacent propellers	27 cm
*a*	Tilt arm length	5 cm
$d_{P_{i}H_{i}}^{A_{i}}$	Position of the propeller with respect to the hinge	[−*a*,0,−1]^*T*^cm
*f*_Σ,*tilt*_	Total thrust to tilt the propellers at hover	13 N
*f*_Σ,*untilt*_	Total thrust to untilt the propellers at hover	2.5 N
*f*_Σ, max_	Maximum total thrust	18 N
*f*_max_	Maximum individual propeller thrust	4.5 N
*f*_min_	Minimum individual propeller thrust	0 N

**FIGURE 7:**
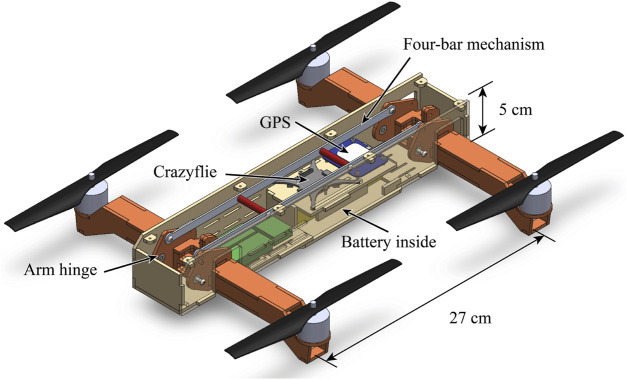
The experimental vehicle frame with the top cover plate taken off. The fuselage shown in beige is constructed with laser-cut wood. The arms shown in red orange are allowed to rotate around the arm hinges, and the rotations of the front and rear arms are coupled by a four-bar mechanism. Round stand-offs shown in red are installed on the long connecting rod of the four-bar mechanism to prevent buckling. Slots are cut on the fuselage for zip-tying wires and other electronics including the ESCs shown in green.

Figure 7 shows the experimental vehicle frame with the top cover plate taken off. We used laser-cut plywood to construct the overall frame because it allows for fast fabrication and provides high-enough precision. The plywood also shows high strength and rigidity which is desirable for aerial vehicles. The overall size of the vehicle frame is chosen to match the area span by the four propellers for compactness.

### 3.3 Tilt angle

Next, we need to choose a tilt angle. The tilt angle mainly affects three vehicle performance indicators, including the maximum flight speed, high-speed agility, and pitch agility. We will first formulate how we can compute these vehicle performance indicators using the aerodynamics model from Section 2.3. Then, we will use the experimental vehicle’s frame parameters to evaluate the vehicle performance and decide the tilt angle and the remaining vehicle parameters in Section 3.4.

#### 3.3.1 Maximum cruise speed

A regular quadcopter is usually not able to achieve a high top speed because it must tilt its body toward the forward flight direction, which increases the area subject to air resistance. This further increases the drag force and requires the propellers to produce even more thrust. However, our proposed vehicle is able to reduce the tilt angle of the central body and therefore could fly at a higher speed given the same hardware limit.

The relationship between the maximum speed and the corresponding designed tilt angle *β* can be solved given the limitation on the vehicle hardware performance. While the vehicle hardware performance can be limited by a range of factors, including the propeller structural strength, ESC current rating, *etc.*, and is dependent on the vehicle speed and other external influences, we will assume that all of these can be generalized to a maximum total thrust of the vehicle *f*_Σ, max_. The correlation between *v*_max_ and *β* can be solved by maximizing *v*_max_ under the following constraints:

$$\text{Cruise dynamics}:\left\{\begin{array}{c} f_{\Sigma }\sin\left(-\alpha +\beta \right)=\frac{1}{2}C_{D}\left(\alpha \right)\rho Av_{\max}^{2}\;\\ f_{\Sigma }\cos\left(-\alpha +\beta \right)=m_{\Sigma }g-\frac{1}{2}C_{L}\left(\alpha \right)\rho Av_{\max}^{2}\; \end{array}\right.$$ (22)

$$\text{Thrust limit}:\left\{\begin{array}{c} f_{\Sigma }\leq f_{\Sigma ,\max}\; \end{array}\right.$$ (23)

The solution to this problem for our experimental vehicle frame is provided in Section 3.4.

#### 3.3.2 High-speed agility

When a quadcopter flies at the maximum cruise speed, all of the thrust capacity is used to counter the vehicle weight and the drag, meaning that it cannot maneuver in any other manner, e.g. accelerate laterally to avoid an obstacle, without falling and reducing its speed. However, if the propellers are allowed to tilt, due to the reduction in drag, the vehicle will no longer saturate its thrust at the same cruise speed. This enables a greater portion of the vehicle’s thrust capacity to be used for maneuvering instead of merely countering drag at high speed, and will improve the high-speed agility of the vehicle. To quantify the agility of the proposed vehicle, we will consider an obstacle-avoidance example. For analysis, we will consider the following simplified maneuvers of the vehicle:

1. Cruise stage
  a. Cruises at a maximum constant speed of *v*_*avoid*_ in the Earth *x*-direction ***x***_*E*_,
2. Turning stage
  a. The vehicle detects an obstacle at a distance *S* in front of it, and starts a turning maneuver,
  b. Constant maximum positive roll torque *τ*_*x*, max_ around the roll axis of the arm frame ***x***_*A*_, and constant maximum pitch torque *τ*_*y*, max_ around the pitch axis of the arm frame ***y***_*A*_ for time Δ*t*,
  c. Constant maximum negative roll torque − *τ*_*x*, max_ around the roll axis of the arm frame ***x***_*A*_, and constant maximum pitch torque − *τ*_*y*, max_ around the pitch axis of the arm frame ***y***_*A*_ for time Δ*t*,
3. Lateral acceleration stage
  a. The roll and pitch torques will change the orientation of the vehicle to allow it to accelerate laterally in the Earth *y*-direction to avoid the obstacle, while maintaining the height and *x*-direction speed of the vehicle.
  b. By the time the x-coordinate of the vehicle reaches the obstacle, the vehicle makes a minimum of *C* *y*-direction clearance with the obstacle.

**FIGURE 8:**
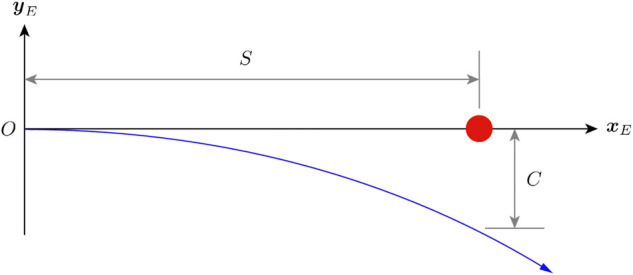
An example obstacle avoidance maneuver. The red dot represents the obstacle and the blue curve represents the flight path. The vehicle is initially flying toward the obstacle and starts an avoidance trajectory at *O* once it detects the obstacle. By the time the x-position of the vehicle reaches *S*, it must make a minimum of *C* y-direction clearance with the obstacle.

Figure 8 shows the example obstacle avoidance maneuver. The maximum crash-free cruise speed *v*_*avoid*_ thus reflects the high-speed agility of the vehicle. The faster a vehicle can travel without having to crash into the obstacle, the more agile it is. The correlation between *v*_*avoid*_ and *β* can be solved by maximizing *v*_*avoid*_ given the following constraints:

$$\text{Cruise dynamics}:\left\{\begin{array}{c} f_{\Sigma ,-}\sin\left(-\alpha_{-}+\beta \right)=\frac{1}{2}C_{D}\left(\alpha_{-}\right)\rho Av_{\mathrm{avoid}}^{2}\;\\ f_{\Sigma ,-}\cos\left(-\alpha_{-}+\beta \right)=m_{\Sigma }g-\frac{1}{2}C_{L}\left(\alpha_{-}\right)\rho Av_{\mathrm{avoid}}^{2}\; \end{array}\right.$$ (24)

$$\text{Turning dynamics}:\left\{\begin{array}{c} \phi =\frac{\tau_{x,\max}}{I_{xx}}\cos\beta \Delta t^{2}\;\\ \psi =-\frac{\tau_{x,\max}}{I_{zz}}\sin\beta \Delta t^{2}\;\\ \alpha_{+}=\alpha_{-}-\frac{\tau_{y,\max}}{I_{yy}}\Delta t^{2}\; \end{array}\right.$$ (25)

$$\begin{array}{ll} \text{Fixed height \& speed} & :\left\{\begin{array}{c} f_{\Sigma ,+}\left(\sin\left(-\alpha_{+}+\beta \right)\cos\phi \cos\psi +\sin\phi \sin\psi \right)=\frac{1}{2}C_{D}\left(\alpha_{+}\right)\rho Av_{\mathrm{avoid}}^{2}\;\\ f_{\Sigma ,+}\left(\cos\left(-\alpha_{+}+\beta \right)\cos\phi \right)=m_{\Sigma }g-\frac{1}{2}C_{L}\left(\alpha_{+}\right)\rho Av_{\mathrm{avoid}}^{2}\cos\phi \; \end{array}\right. \end{array}$$ (26)

$$\text{Minimum clearance}:\left\{\begin{array}{c} s_{y}\left(t=\frac{S}{v_{\max}}\right)\leq -C\; \end{array}\right.$$ (27)

$$\text{Torque capacity limit}:\left\{\begin{array}{c} \tau_{y,\max}=l\delta \left(4f_{\max}-f_{\Sigma ,+}\right)\cos\beta \;\\ \tau_{x,\max}=l\left(1-|\delta |\right)\left(4f_{\max}-f_{\Sigma ,+}\right)\; \end{array}\right.$$ (28)

Where $\left(\phi ,\psi \right)$ are the roll and yaw angles of the vehicle after the turning stage, $\left(f_{\Sigma ,-},f_{\Sigma ,+}\right)$ are the total vehicle thrusts before and after the turning stage, $\left(\alpha_{-},\alpha_{+}\right)$ are the angles of attack before and after the turning stage, and *δ* ∈ [0, 1] is the fraction of the vehicle’s torque capacity used to produce a pitch torque. Note that although the vehicle produces a roll torque around the roll axis of the arm frame *A*, the yaw angle of the vehicle will also change. This is because the tilted propellers will produce a torque around the yaw axis of the central body frame *C*. For the given turning maneuver, the total thrust of the vehicle will monotonically increase from *f*_Σ,−_ at *t* = 0, to *f*_Σ,+_ at *t* = 2Δ*t*, and stay at *f*_Σ,+_ for the remainder of the flight. Throughout the turning stage, since the magnitudes of the desired roll and pitch torques are constant, the magnitude of the thrust shift across the four propellers to generate the desired roll and pitch torques are also constant. As a result, at *t* = 2Δ*t* where the total thrust is the highest, the thrust shift will result in one propeller having the peak thrust. We limit such peak thrust to *f*_max_ and with some algebraic manipulation, we can find the torque limit as shown by Eq. 28. The solution to this problem is highly dependent on the vehicle’s dynamic properties and is provided for our experimental vehicle frame in Section 3.4.

#### 3.3.3 Pitch agility near hover

The change in tilt angle *β* changes the maximum pitch torque *τ*_*y*_ that the vehicle can generate. This is because when the arms tilt, the moment arm between the front rotors’ thrust axes and the rear rotors’ thrust axes changes. Assuming that near hover, the maximum thrust difference between the front rotors and the rear rotors is Δ*f*, the maximum pitch torque is thus *τ*_*y*, max_ = Δ*fl* cos *β*. We note that this torque reduces as the tilt angle increases. Nevertheless, this problem can be mitigated by designing the rear rotors to be higher than the front rotors with respect to the central body. For a rotor height offset of Δ*h*, the maximum pitch torque now becomes *τ*_*y*, max_ = Δ*f*(*l* cos *β* + Δ*h* sin *β*). However, this results in an increase in vehicle height, which restricts the vehicle’s capability to maneuver in limited space. In addition, the drag area may also increase if the height offset is achieved by simply skewing the vehicle frame. Therefore, the designer will need to consider the application to find a balance between maximum pitch torque, the height of the vehicle, and the other vehicle performance indicators.

It is important to note that as the vehicle speed increases, the maximum thrust difference Δ*f* will decrease due to additional drag on the vehicle. As discussed in the previous section, at high speed, because tilting the arms reduces the drag, Δ*f* will be relatively larger when the arms are tilted, which can lead to a relatively higher maximum pitch torque. Therefore, while tilting the arms always reduce the pitch agility of the vehicle near hover, it does not necessarily reduce the pitch agility at higher speed.

### 3.4 Experimental vehicle tilt angle design

Using the parameters of the experimental vehicle frame in Table 1, we can solve for the correlations between the tilt angle *β* and the three vehicle performance indicators above. For quantifying the high-speed vehicle agility, we set the detection range to *S* = 10 m and clearance to *C* = 1 m.

**FIGURE 9:**
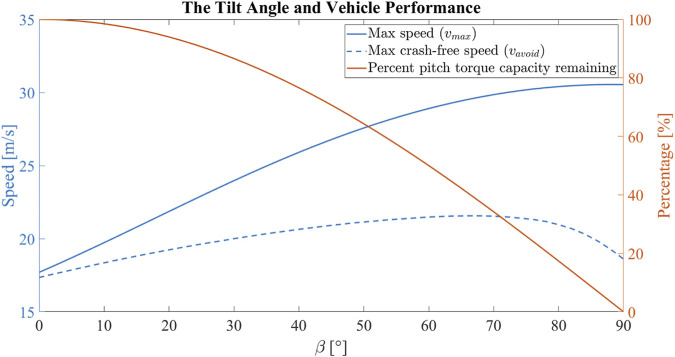
The correlation between the tilt angle and the vehicle performance for the experimental vehicle. Increasing the tilt angle increases the maximum cruise speed of the vehicle with a decreasing marginal gain. Increasing the tilt angle increases the maximum crash-free cruise speed of the vehicle up until *β* = 68°. However, increasing the tilt angle decreases the pitch torque capacity.

Figure 9 shows how the tilt angle changes the max linear speed *v*_max_, the max crash-free speed *v*_*avoid*_, and the remaining pitch torque capacity as compared to the torque at zero tilt angle. Increasing the tilt angle increases the maximum cruise speed of the vehicle with a decreasing marginal gain. The maximum speed that the vehicle can achieve is 33.28 m s^−1^ at a designed tilt angle of 88.2°, which is a 64.8% increase from the maximum speed of 20.19 m s^−1^ when the tilt angle is zero. The maximum crash-free cruise speed of the vehicle increases as the tilt angle increases, but is maxed out at *β* = 68°. The maximum crash-free cruise speed that the vehicle can achieve is 21.56 m s^−1^ at a designed tilt angle of 68°, which is a 24.3% increase from the maximum speed of 17.35 m s^−1^ when the tilt angle is zero. However, increasing the tilt angle decreases the pitch torque capacity at hover monotonically.

In the end, we have chosen a tilt angle of *β* = 20° to preserve much of the pitch torque capacity, while creating enough differences to be observed in the maximum speed and maximum crash-free speed so that we can validate the analyses results with experiments. The 20° tilt angle is predicted to increase the maximum speed of the vehicle from 20.19 m s^−1^ to 24.70 m s^−1^, and the maximum crash-free speed of the vehicle for the given trajectory from 17.35 m s^−1^ to 19.24 m s^−1^. On the other side, the reduction in maximum pitch torque at hover is 6.03% in the tilted configuration.

Lastly, we will need to choose the spring and the anchoring points to produce the desired tilt and untilt thrusts. The spring force, anchoring points, and the desired tilt/untilt thrusts are correlated by Eq. 21, and the standard spring equation $f_{s_{i}}=-k\left(\|d_{S_{i}M_{i}}\|-l_{0}\right)\frac{d_{S_{i}M_{i}}}{\left\|d_{S_{i}M_{i}}\right\|}$ . We approach this problem by first experimentally determining the spring constants for a set of springs in stock that will fit in the vehicle frame. Then, we compute the exact anchoring points for all the springs by numerically solving the full equations with additional space constraints. Lastly, we choose the spring and the corresponding anchoring points that would minimize the size of the tilting mechanism. The vehicle’s tilt angle and all other relevant properties are summarized in Table 2.

**TABLE 2:** Experimental vehicle tilting-related properties.

Symbol	Parameter	Value
*β*	Tilt angle	20°
*l*_0_	Spring rest length	1.75 cm
*k*	Spring constant	8 N cm^−1^
$d_{M_{i}H_{i}}^{A_{i}}$	Position of spring end 1 with respect to the hinge	[−4, 0, 1]^*T*^cm
$d_{S_{i}H_{i}}^{A_{i}}$	Position of spring end 2 with respect to the hinge	[1.3, 0, −1]^*T*^cm

To ensure consistent configuration transition in actual flight, it is important to keep the springs under the limit of proportionality to prevent degradation. For long-term use, fatigue analysis on the springs is desired. In addition, because the wires powering the rotors will pass around the arm hinges, it is crucial to minimize the friction that wires introduce to the quadcopter arms by using softer wires, running cables properly, *etc.* It is also worth noting that the use of springs is rather a design choice but not the only option. In the end, our goal is to put a larger torque on the quadcopter arm in the untilted configuration, and a smaller torque on the quadcopter arm in the tilted configuration. Therefore, other solutions like using magnets of different strengths to attract the quadcopter arms can be applied, and may even offer a longer life cycle and smaller size.

With the design of the experimental vehicle finalized, we will now validate its capabilities with experiments.

## 4 Experimental validation

In this section, we will use the experimental vehicle to validate the capabilities of the proposed design, including *1*) the reliability of the tilting mechanism, *2*) the improvement in the top linear speed, *3*) the improvement in the high-speed agility, and 4) the increase in the energy efficiency.

### 4.1 Experiment setup

For all of our tests, we fly the vehicle outdoors in a flat grass field at the Richmond Field Station, Richmond. All the speed measurements are ground speeds, and while we do not specifically characterize the influence of wind, we strive to ensure consistency in the experimental results by 1) conducting experiments only when the wind is low, 2) conducting experiments in a short time frame to minimize wind variation, and 3) flying the vehicle consistently in the same direction.

The vehicle is localized by fusing readings from the following sensors:

1. Inertial measurement unit (accelerometer and rate-gyroscope) running at 500 Hz,
2. 3-axis magnetometer running at 100 Hz,
3. Global positioning system running at 5 Hz.

The sensor readings are fused *via* an off-the-shelf extended Kalman filter (EKF) algorithm taken from the open-source PX4 firmware (Meier et al., 2015). The IMU and magnetometer are a part of the flight controller and the GPS is connected to the flight controller *via* a serial port (UART). The EKF is run on the flight controller at 500 Hz, predicting the states forward using the IMU data, and using the GPS and magnetometer readings for the correction step of the EKF. The state estimates are then used by the flight controller for closed-loop control.

Data from the above sensors and the state estimates are logged *via* radio at 100 Hz for post-processing. Additionally, the voltage and current readings from the battery are measured using a power module and are also logged to calculate the power consumption of the quadcopter in the untilted and tilted configurations.

**FIGURE 10:**
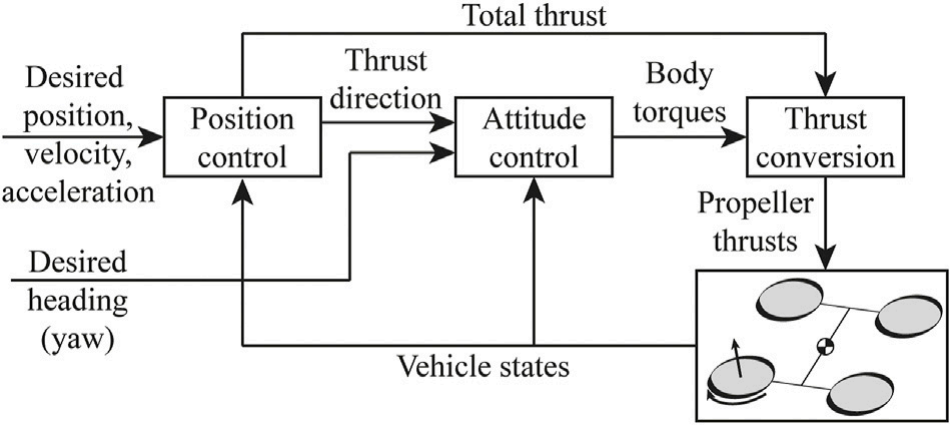
Block diagram of the quadcopter controller.

The quadcopter is controlled autonomously and tracks the desired position, velocity, acceleration, and yaw angle by using a cascaded position and attitude controller as shown in Figure 10.

The position and attitude controller computes the desired body torques $\tau^{A}={\left[\tau_{x},\tau_{y},\tau_{z}\right]}^{T}$ and total thrust *f*_Σ_ in the combined arm *A* frame required to track the desired thrust direction and the desired yaw angle. Individual rotor thrusts $u={\left[f_{p_{1}},f_{p_{2}},f_{p_{3}},f_{p_{4}}\right]}^{T}$ required to generate the desired total thrust and the desired body torques are then computed using the following mapping:

$$u=\left[\begin{array}{c} f_{p_{1}}\\ f_{p_{2}}\\ f_{p_{3}}\\ f_{p_{4}} \end{array}\right]={\left[\begin{array}{c} M_{f_{\Sigma }}\\ M_{\tau^{A}} \end{array}\right]}^{-1}\left[\begin{array}{c} f_{\Sigma }\\ \tau_{x}\\ \tau_{y}\\ \tau_{z} \end{array}\right]=M^{-1}\left[\begin{array}{c} f_{\Sigma }\\ \tau^{A} \end{array}\right]$$ (29)

Where $M_{f_{\Sigma }}\in R^{1\times 4}$ is the mapping from ***u*** to *f*_Σ_, $M_{\tau^{A}}\in R^{3\times 4}$ is the mapping from ***u*** to ***τ*** ^*A*^, and $M\in R^{4\times 4}$ is the combined mapping. The mapping is computed using the geometry of the vehicle and the torque $\tau_{p_{i}}$ from each propeller which correlates to the thrust $f_{p_{i}}$ by $\tau_{p_{i}}={\left(-1\right)}^{i}\kappa f_{p_{i}}$ , where *κ* is the thrust to torque coefficient of the propeller. Since the body torques and the desired total thrust are in the combined arm frame *A*, the entries for the mapping matrices are given as:

$$M_{f_{\Sigma }}=\left[\begin{array}{cccc} 1 & 1 & 1 & 1 \end{array}\right]$$ (30)

$$M_{\tau^{A}}\left[:,i\right]=S\left(R^{AC}d_{P_{i}C}^{C}\right)z_{A}^{A}+{\left(-1\right)}^{i}\kappa z_{A}^{A}$$ (31)

Lastly, we can compute the combined mapping matrix ***M*** for the untilted and tilted configurations:

$$M_{\mathrm{untilted}}=\left[\begin{array}{cccc} 1 & 1 & 1 & 1\\ -\frac{l}{2} & \;-\frac{l}{2} & \frac{l}{2} & \frac{l}{2}\\ -\frac{l}{2} & \frac{l}{2} & \frac{l}{2} & \;-\frac{l}{2}\\ -\kappa & \kappa & \;-\kappa & \kappa \end{array}\right]$$ (32)

$$M_{\mathrm{tilted}}=\left[\begin{array}{cccc} 1 & 1 & 1 & 1\\ -\frac{l}{2} & \;-\frac{l}{2} & \frac{l}{2} & \frac{l}{2}\\ -\cos\beta \left(a+\frac{l}{2}\right)+a & \cos\beta \left(a-\frac{l}{2}\right)+a & \cos\beta \left(a-\frac{l}{2}\right)+a & \;-\cos\beta \left(a+\frac{l}{2}\right)+a\\ -\kappa & \kappa & \;-\kappa & \kappa \end{array}\right]$$ (33)

**FIGURE 11:**
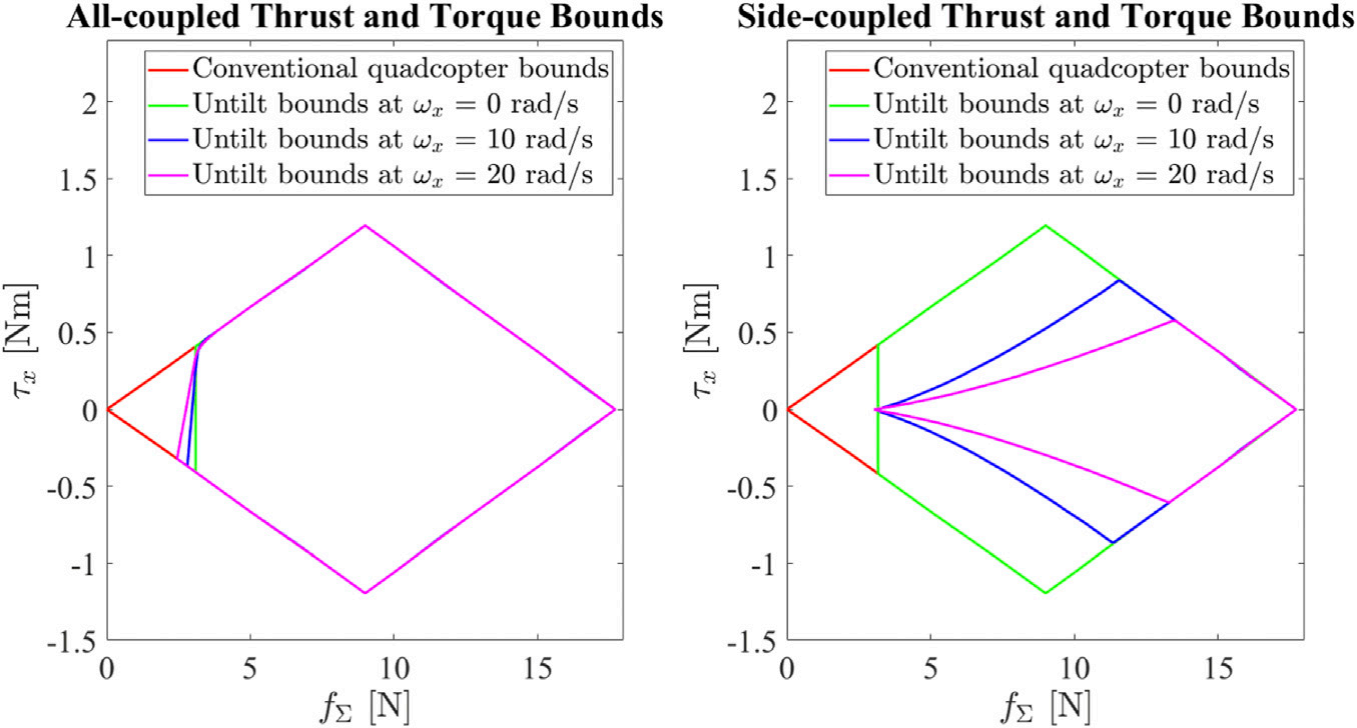
The limit on the roll torque and the total thrust that the vehicle can produce in the side-couple and all-coupled configurations for different rolling speeds, to prevent the arms from untilting in the tilted configuration.

Combining this with the thrust bounds we computed in Section 2.4, we can find the limit on the total thrust and the desired body torques. The vehicle in the all-coupled configuration has the highest agility. The pitch torque capacity is higher, and the thrust bounds are almost not affected by the motion of the vehicle. The vehicle in the side-coupled has a lower maximum roll and yaw torque when the rolling speed is high. As an example, Figure 11 shows the limit on the roll torque and the total thrust that the vehicle can produce in the side-couple and all-coupled configurations for different rolling speeds, to prevent the arms from untilting in the tilted configuration.

As compared to a conventional quadcopter with the same vehicle parameters without the ability to tilt, the tilt-rotor has tighter bounds on the torque thrust and roll torque to prevent untilting. At zero rolling speed, the untilt bounds are identical for the all-coupled and side-coupled configurations. As the rolling speed increases, the gyroscopic torque discussed in Section 3.1 comes into play, and tightens the bounds for the vehicle in the side-coupled configuration. However, in the all-coupled configuration, the bounds are effectively not affected at all, and the vehicle maintains the same agility regardless of the maneuver. As a result, we have kept our vehicle in the all-coupled configuration, and we have found that the vehicle is able to maintain its configuration without any programmed tilt/untilt thrust and torque bounds.

### 4.2 Experiments

#### 4.2.1 Changing configuration test

**FIGURE 12:**
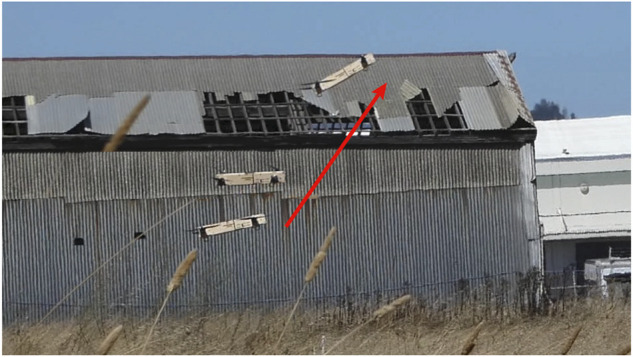
The vehicle switches from the untilted to the tilted configuration. An offset is added to the desired position to accommodate for the change in the vehicle position due to the sudden high thrust.

The transition between the tilted and untilted configurations is tested. The transition from the untilted configuration to the tilted configuration is accomplished by commanding a high total thrust for a fraction of a second. Right after the morphing, the same controller before the transition resumes to function but is updated to use the tilted mapping matrix *M*_*tilted*_. To accommodate for the change in the vehicle position from suddenly producing a high thrust, we add an offset to the desired position right after the morphing. Figure 12 shows the vehicle switching from the untilted to the tilted configuration.

**FIGURE 13:**
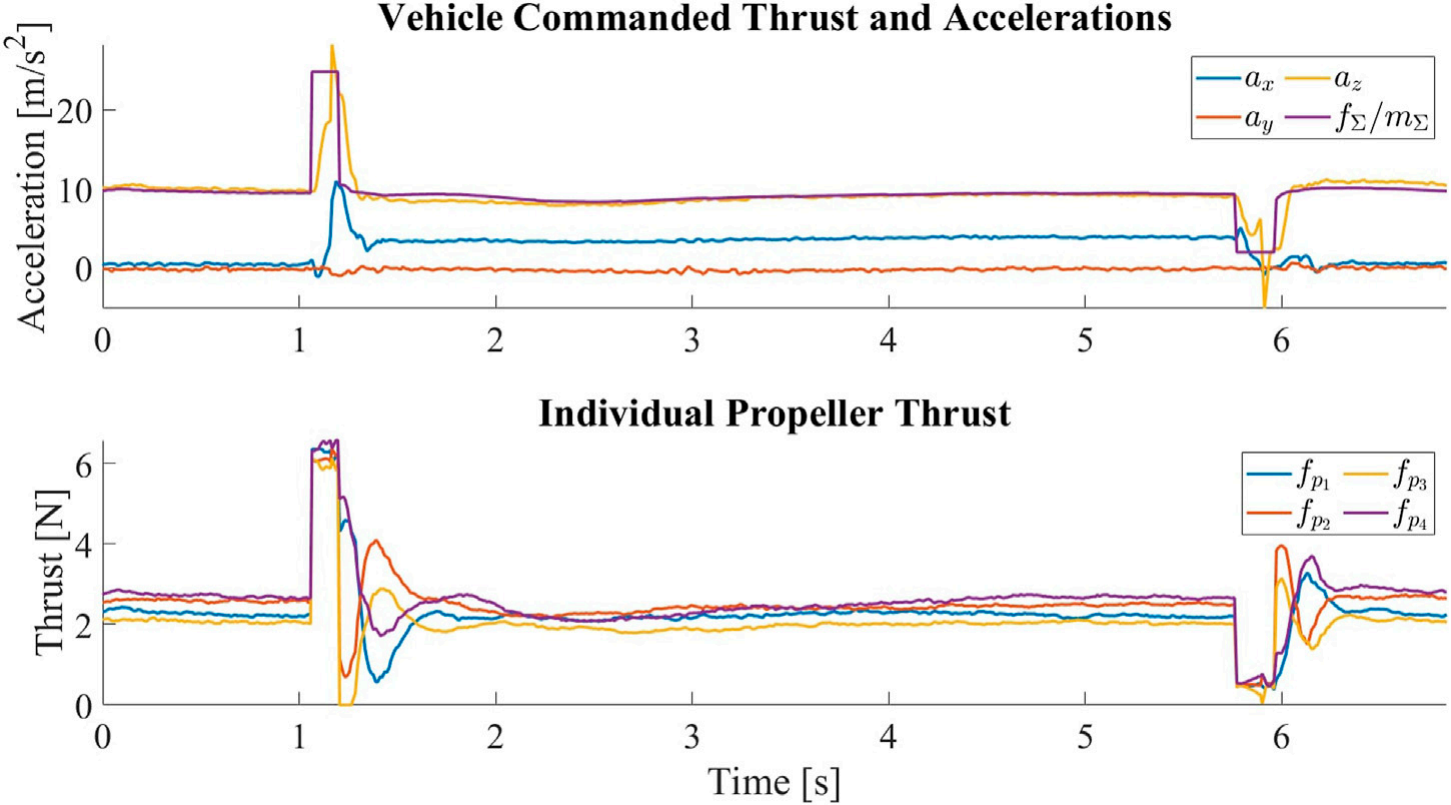
The vehicle commanded thrust normalized by the vehicle mass and the measured accelerations in the central body frame C for one tilt and untilt cycle. At around t = 1s, the vehicle is commanded to tilt by producing a sudden high thrust. The surge in thrust is followed by a surge in the acceleration along *z*_*C*_, which is then followed by an increase in the acceleration along *x*_*C*_, meaning that the thrust axes of the propellers have been tilted forward. The negative *x*_*C*_ acceleration between transitions indicates the change of the vehicle’s pitch angle such that the propellers are pointing upward to keep the vehicle at hover. At around *t* = 4.5s, the vehicle is commanded to untilt by producing a sudden low thrust. The drop in thrust is followed by a drop in the acceleration along *z*_*C*_, which is then followed by a drop in the magnitude of acceleration along *x*_*C*_, meaning that the thrust axes of the propellers have been restored. Despite the change in the mapping matrix, we can see that the individual propeller thrusts are very close once the vehicle has stabilized after the transition.

To switch back to the untilted configuration, we simply command a near zero thrust for a fraction of a second. Right after the morphing, the controller is switched back to use the untilted mapping matrix *M*_*untilted*_. The sudden loss of thrust causes the vehicle to fall, so an opposite offset is added to the desired position to accommodate for the change in the vehicle position. The tilting and untilting are repeated 20 times and show no signs of failure. Figure 13 shows the vehicle commanded thrust and the measured accelerations for one tilt and untilt cycle.

#### 4.2.2 Maximum linear speed tests

The maximum speed of the vehicle is tested by flying the vehicle in a straight line in the following manner:

1. Accelerate at a constant linear acceleration of *a*,
2. Check if the maximum total thrust *f*_Σ, max_ is reached, if so start decelerating until rest.
3. Record the maximum speed that the vehicle has reached *v*_max_.

Since the maximum total thrust *f*_Σ, max_ is above the tilt thrust *f*_Σ,*tilt*_, we bolted the tilting mechanism in the untilted configuration to imitate a vehicle without the ability to tilt. In order to prevent the vehicle from flying beyond the flight space, we choose *a* to be 3.125 m s^−2^. Adding the acceleration term to Eq. 22, we can predict that the maximum speed in the untilted configuration is 17.70 m s^−1^, and the maximum speed in the tilted configuration is 21.86 m s^−1^. The actual experiment is repeated three times for each configuration. The experimental results are summarized in Table 3.

**TABLE 3:** Maximum linear speed achieved by the vehicle and the associated angle of attack.

	Untilted		Tilted	
Trial	Max speed	Max angle of attack	Max speed	Max angle of attack
1	18.65 m s^−1^	−41.47°	20.81 m s^−1^	−25.79°
2	18.64 m s^−1^	−45.20°	21.59 m s^−1^	−16.05°
3	19.05 m s^−1^	−39.73°	21.02 m s^−1^	−26.59°
Average	18.77 m s^−1^	−42.13°	21.14 m s^−1^	−22.81°
Standard deviation	0.24 m s^−1^	2.79°	0.40 m s^−1^	5.87°

We can see that the average maximum speed of the vehicle in the tilted configuration is 12.5% higher than in the untilted configuration, and the results are repeatable. We do note that the vehicle in the untilted configuration is flying faster than the prediction. We suspect that this has to do with the fact that the lift model assumes that the angle of attack is in the linear region which will show a very high downward lift on the vehicle when the angle of attack is large. However, at this speed, we record that the angle of attack of the vehicle in the untilted configuration is almost − 45°, which is beyond the linear region. As a result, the actual downward lift on the vehicle is smaller than the prediction, meaning that more of the vehicle thrust can be used to counteract the drag, thus allowing the vehicle in the untilted configuration to fly faster.

#### 4.2.3 Obstacle avoidance tests

The high-speed agility of the vehicle is tested by having the vehicle track the obstacle avoidance trajectory discussed in Section 3.3.2. We create an imaginary obstacle on our path with *S* = 10 m, and command the vehicle to cruise at the computed *v*_max_ and then turn to avoid the obstacle to achieve a clearance of *C* = 1 m. We limit the individual propeller thrust at *f*_max_, and compare the actual flight speed and clearance with the commanded ones to evaluate the real agility of the vehicle. The experimental results are summarized in Table 4.

**TABLE 4:** The actual flight speed and clearance and the commanded flight speed and clearance.

Configuration	Commanded speed	Actual speed	Commanded clearance	Actual clearance
Untilted	17.35 m s^−1^	17.70 m s^−1^	1 m	1.03 m
Tilted	19.24 m s^−1^	19.03 m s^−1^	1 m	1.24 m

We can see that given the same thrust constraint, the vehicle is able to achieve the commanded clearance of *C* = 1 m in both the untilted and tilted configurations, and can reach a higher flight speed without crashing in the tilted configuration.

#### 4.2.4 Aerodynamic performance tests

The reduction in drag allows less thrust to be produced to travel at the same speed, which increases the energy efficiency of the vehicle. To test the aerodynamic performance, the vehicle is flown at commanded horizontal speeds *v*_*des*_ of $\left\{10.0,12.5,15.0,17.5,20.0\right\}$ m s^−1^ in a straight line in the following manner:

1. Accelerate from rest to the cruising speed *v*_*des*_ over a specified acceleration distance *s*_accel_,
2. Cruise at *v*_*des*_ over a specified cruise distance *s*_cruise_,
3. Decelerate from cruising speed to rest over a specified deceleration distance *s*_decel_.

Voltage and current data collected from the power module is evaluated over the steady state of the cruising portion of the trajectory, which is selected to last 5 s to get approximately 500 data points.

**FIGURE 14:**
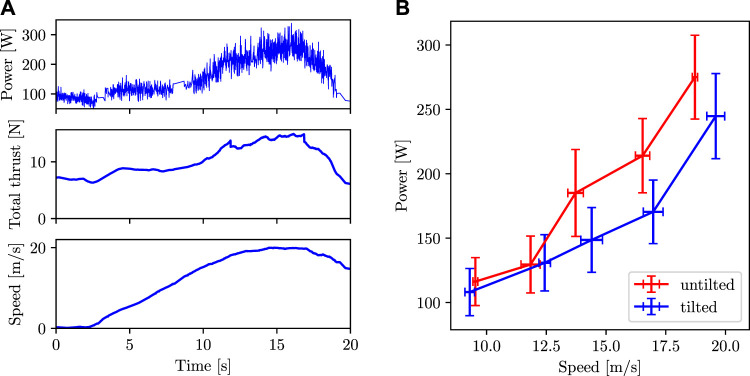
**(A)** Power, total thrust, and speed vs time for a single experiment. Data in this plot is from the experiment where the quadcopter is commanded to fly in the tilted configuration at 20 m s^−1^. **(B)** Power consumption vs speed when flying in untilted and tilted configurations. The data points are the average values and the error bars represent one standard deviation in the data.

A sample plot of power and speed vs time is shown in Figure 14A. This specific plot is for the case of the quadcopter commanded to fly in the tilted configuration at 20 m s^−1^.

The plot of average power vs average speed is shown in Figure 14B. The power consumption is lower in the tilted configuration than in the untilted configuration at high speed. We can see that the power consumption in the tilted configuration in the speed range of 15–20 m s^−1^ is more than 20% lower as compared to the untilted configuration.

## 5 Conclusion

In this paper, we have presented a novel quadcopter design capable of tilting the propellers into the forward flight direction in mid-air to reduce the drag without the use of additional actuators. The reduction in drag allows the vehicle to fly at a higher top speed with higher agility, and improves the flight efficiency at high speed. Unlike the other multirotor–fixed-wing combo quadcopters, the proposed vehicle does not have wings. While this sacrifices the cruise efficiency, the vehicle has higher agility as the area subject to aerodynamic forces is kept small. Especially, the vehicle will not have high drag during rolling motion due to large wings paddling in the air like fixed-wing vehicles. By using simple sprung hinges instead of actuators or other complex mechanisms, the design is thus relatively less complicated than other aerial morphing vehicles. On the other side, the use of a passive tilting mechanism means that the arms can only be tilted in one direction with a fixed tilting angle, and cannot achieve the arbitrary attitude of other actively tilted quadcopters.

The dynamics of such a vehicle were derived. Based on the dynamics, we discussed the key design parameters including the tilt angle and the vehicle configuration. The effects that these parameters have on the vehicle performance are presented, and the relevant design trade-offs are discussed. Analyses show that while the vehicle is always less agile near hover as compared to a conventional vehicle due to the introduction of additional thrust bounds, it does have a higher top speed and higher agility at high speed as lesser thrust capacity is used to counteract the aerodynamic forces in the tilted configuration.

An experimental vehicle with an overall size similar to a regular quadcopter is built to validate the analyses. Experiments are done to validate the capabilities of the vehicle. First, the vehicle is shown to transition between the tilted and untilted configurations reliably. Then, the vehicle is shown to have reached a higher maximum linear speed under the same thrust limit in the tilted configuration. Furthermore, the vehicle is shown to be more agile at high speed, as it can fly faster while avoiding a defined obstacle in the tilted configuration. Finally, the vehicle is shown to have a better energy efficiency than a conventional quadcopter at a higher speed.

The proposed design is thus able to fly at a higher top speed (by 12.5%), has higher high-speed agility (by 7.5%) and higher efficiency (20% lower power consumption for a speed range of 15–20 m s^−1^) with little trade-offs in mechanical complexity and low-speed agility. This can be useful for applications that are time-sensitive, such as package delivery and drone racing. In the future, the vehicle can be designed such that the tilt angle can be easily reconfigured, allowing it to fit a wide range of applications. The frame of the vehicle can also be designed to be more aerodynamically efficient, allowing for an even higher top speed and better high-speed agility. One approach to improve the aerodynamic efficiency of the vehicle frame is to reduce its vertical dimension, which can be achieved by reducing the size of the tilting mechanism through the use of shorter but stronger springs. In addition, the vehicle frame can be designed like an airfoil shape to reduce the drag coefficient, and even generate lift to counteract the vehicle weight.

## Data Availability

The raw data supporting the conclusion of this article will be made available by the authors, upon reasonable request.
