# Corrigendum: QUaRTM: A Quadcopter with Unactuated Rotor Tilting Mechanism capable of faster, more agile, and more efficient flight

**DOI:** 10.3389/frobt.2023.1199090

**Published:** 2023-04-19

**Authors:** Jerry Tang, Karan P. Jain, Mark W. Mueller

**Affiliations:** High Performance Robotics Lab, Department of Mechanical Engineering, University of California, Berkeley, Berkeley, CA, United States

**Keywords:** morphing quadcopter, agile, efficient, high-speed, mechanism design (MD), aerodynamics

We would like to address three issues identified in our publication. Specifically, we have made necessary corrections to errors present in tables relating to vehicle properties, rectified an error in a figure pertaining to an outdoor experiment, and included additional information on wind conditions during experiments. We regret for our oversight of these issues in the original article and want to emphasize that the scientific conclusions drawn in the article remain unaffected. The original article has been updated.

In the published article, there were errors in **Table 1** Experimental vehicle frame properties and **Table 2** Experimental vehicle tilting-related properties, as published. A structural change was made to the experimental vehicle to improve its modularity, and several properties were changed but were not updated. The corrected vehicle properties are as follows: *a* = 5 cm, *k* = 8 N cm^−1^, $d_{M_{i}H_{i}}^{A_{i}}$ = [−4,0,1]^*T*^cm, and $d_{S_{i}H_{i}}^{A_{i}}$ = [1.3,0,−1]^*T*^cm.

In addition, there was an error in Figure 13, which shows the vehicle commanded thrusts and the measured accelerations. Data from an indoor flight test were accidentally used, and the coordinate transformation matrix used to change the IMU reference frame was not transposed, resulting in a negative ***x***_*C*_ acceleration when the propellers are tilted forward, as shown by the plot, whereas the acceleration should be positive. A corrected plot with outdoor flight data and the right acceleration values is shown in the following figure. The description has also been updated to reflect the changes.

**FIGURE 13:**
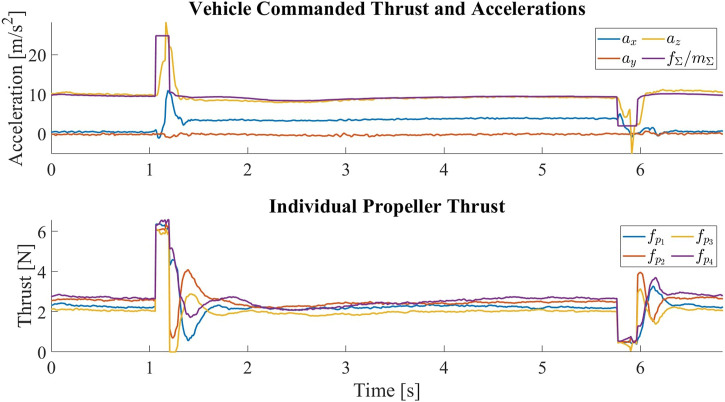
The vehicle commanded thrust normalized by the vehicle mass and the measured accelerations in the central body frame *C* for one tilt and untilt cycle. At around *t* =1*s*, the vehicle is commanded to tilt by producing a sudden high thrust. The surge in thrust is followed by a surge in the acceleration along ***z***_*C*_, which is then followed by an increase in the acceleration along ***x***_*C*_, meaning that the thrust axes of the propellers have been tilted forward. The negative ***x***_*C*_ acceleration between transitions indicates the change of the vehicle’s pitch angle such that the propellers are pointing upward to keep the vehicle at hover. At around *t* =4.5*s*, the vehicle is commanded to untilt by producing a sudden low thrust. The drop in thrust is followed by a drop in the acceleration along ***z***_*C*_, which is then followed by a drop in the magnitude of acceleration along ***x***_*C*_, meaning that the thrust axes of the propellers have been restored. Despite the change in the mapping matrix, we can see that the individual propeller thrusts are very close once the vehicle has stabilized after the transition.

The authors would also like to add information on the wind condition in the first paragraph of 4.1 Experiment setup. The corrected paragraph is given as follows:

“For all of our tests, we fly the vehicle outdoors in a flat grass field at the Richmond Field Station, Richmond. All the speed measurements are ground speeds, and while we do not specifically characterize the influence of wind, we strive to ensure consistency in the experimental results by 1) conducting experiments only when the wind is low, 2) conducting experiments in a short time frame to minimize wind variation, and 3) flying the vehicle consistently in the same direction.

The vehicle is localized by fusing readings from the following sensors:”

